# Acute and early developmental outcomes of children with Duarte galactosemia

**DOI:** 10.1002/jmd2.12267

**Published:** 2021-12-16

**Authors:** Judith L. Fridovich‐Keil, Grace Carlock, Sneh Patel, Nancy L. Potter, Claire D. Coles

**Affiliations:** ^1^ Department of Human Genetics Emory University School of Medicine Atlanta Georgia USA; ^2^ Emory College Atlanta Georgia USA; ^3^ Department of Speech and Hearing Sciences Washington State University Spokane Washington USA; ^4^ Department of Psychiatry and Behavioral Sciences Emory University School of Medicine Atlanta Georgia USA

**Keywords:** acute outcomes, Duarte galactosemia, early development, early intervention

## Abstract

A recent study demonstrated that children with Duarte galactosemia (DG) do not show increased prevalence of detectable developmental complications when 6–12 years old. However, that study left unanswered whether infants with DG might be at increased risk for acute problems when drinking milk or whether children with DG younger than 6 years might show increased prevalence of perhaps transient developmental challenges. Here, we have addressed both of these questions by analyzing parent/guardian‐reported data collected retrospectively for 350 children, 206 with DG and 144 unaffected siblings from the same families. The variables analyzed included whether each child had experienced (1) acute complications in infancy, (2) early intervention services when <3 years old, and/or (3) special educational services when 3–5 years old. For each case–control comparison, or case‐by‐diet comparison, we used logistic regression that included the following potential covariates: age, sex, race, family income, and parent education, as appropriate. We found that none of the three outcome variables tested showed significant differences between cases and controls, or among cases as a function of galactose exposure in infancy. To the limits of our study, we therefore conclude that regardless of whether a child with DG drinks milk or low‐galactose formula as an infant, they are not at increased risk for acute complications or early childhood developmental challenges that require intervention.

**Synopsis**

A large cohort of children with Duarte galactosemia showed no increased prevalence of parent/guardian‐reported acute complications in infancy regardless of milk exposure and also no increased receipt of early intervention or special educational services when under the age of 6.

## 1 INTRODUCTION

Duarte galactosemia (DG)^1^ is a common allelic variant of the potentially lethal inborn error of metabolism, classic galactosemia.^2^ Infants with DG are detected by newborn screening at a prevalence of up to about 1/4000 births in many states.^3^ Because, as infants, they accumulate abnormal levels of galactose metabolites in blood and urine when consuming a milk diet (breast milk or a cow's milk–based formula), for many decades, some clinicians advised switching these babies from milk to a low‐galactose formula for the 1st year of life, after which the abnormal galactose metabolites self‐resolve regardless of diet.^1^

Recently, we conducted a large observational study of developmental outcomes of children with DG.^4^ For that study, we recruited and enrolled 206 children with DG and 144 unaffected sibling controls, all 6–12 years old. We gathered demographic, general health, and diet information for each child using a parent/guardian survey and also asked retrospective questions about the presence or absence of any acute complications in infancy, early intervention (birth to <3 years old), or special educational services received when 3–5 years old.

All 350 children completed direct assessment of 73 developmental outcomes representing five domains: physical measures, cognitive development, socioemotional development, speech and hearing including auditory processing, and motor skills. Case–control status for each child was confirmed by full *GALT* gene sequencing. As reported previously,^4^ this study found no significant differences between cases and controls in any of the developmental outcomes directly assessed. Because about 40% of cases had consumed milk in their 1st year of life, while the remainder had consumed low‐galactose formula, we further asked whether, among the children with DG, there was any significant association between galactose exposure in infancy and developmental outcomes measured and there was not.

These direct assessments were informative for addressing the potential developmental risks of children with DG in midchildhood (ages 6–12), but they did not address whether these same children might have experienced increased prevalence of acute complications early in infancy or transient developmental challenges in early childhood. Here, we approach these questions using logistic regression to compare the relevant parent/guardian responses for cases and controls. These results substantially extend what is known about outcomes in DG.

## 2 MATERIALS AND METHODS

### 2.1 Study subjects and data collection

The study subjects described here represent the same cohort of 206 cases and 144 controls described in Carlock et al.^4^ All participated following appropriate informed consent (Emory IRB 00081271; PI: JL Fridovich‐Keil). Data regarding child diet, general health, acute outcomes in early infancy, potential covariates (age, sex, race, relative family income, and parent education level), and any interventions received when birth to 3, or 3 to 5, were collected using a parent/guardian survey administered via REDCap.^5^, ^6^

### 2.2 Statistical analyses

We used logistic regression models to evaluate the relationship between DG case–control status, dietary galactose exposure of cases in infancy, and each of three binary outcome measures: presence or absence of acute complications in infancy, receipt or nonreceipt of early intervention when <3 years old, and receipt or nonreceipt of special educational services when 3–5 years old. Specifically, we first regressed each of the three binary outcome variables on case–control status adjusting as needed for effects of age, sex, race/ethnicity, average family income, and parent education. Because >93% of both cases and controls self‐identified as White/non‐Hispanic, we dichotomized the race/ethnicity variable to White/non‐Hispanic versus all other.

To assess the impact of dietary galactose exposure in infancy on outcomes among cases, we applied a similar procedure to regress each binary outcome variable with whether each child drank milk or low‐galactose formula in the 1st year of life, adjusting as needed for the covariates listed above. All analyses were conducted using *R* software packages (https://www.r-project.org).

## 3 RESULTS

### 3.1 Study subjects and data collection

This study utilized data collected from the parent/guardians of 206 children with DG (cases) and 144 unaffected siblings controls. Families were recruited as part of a larger study^4^ with assistance of the newborn screening programs or metabolic clinics that had originally diagnosed the affected children. Families were recruited from 13 states across the continental United States, and cases and controls were well matched for age, sex, race, and socioeconomic factors.^4^ Details of recruiting and participant demographics can be found in Reference 4 and at clinicaltrials.gov (https://clinicaltrials.gov/ct2/show/NCT02519504).

Data about the presence or absence of acute complications in infancy, and receipt or nonreceipt of early intervention services when <3 years old, or special educational services when 3–5 years old, were collected retrospectively using a parent/guardian survey administered online via REDCap. Using this same survey, we also collected information about child diet with respect to galactose exposure in infancy, child general health to rule out possible confounding conditions, and child and family demographic factors to allow adjustment for potential covariates, as appropriate.

### 3.2 Acute outcomes in early infancy

**FIGURE 1:**
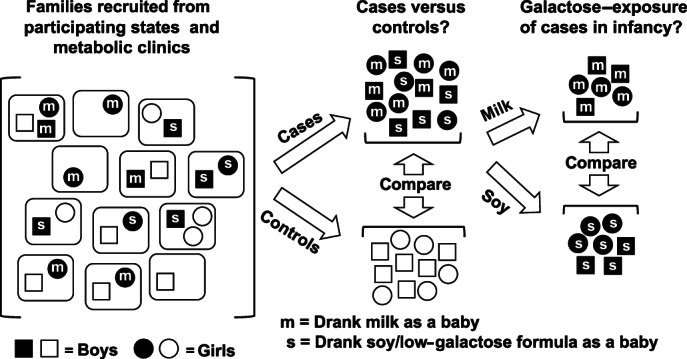
Design of this study. For each of the three outcome measures followed here, (1) presence versus absence of acute complications in infancy, (2) receipt of early intervention services when <3 years old, and (3) receipt of special educational services when 3–5 years old, we first compared prevalence between cases (shaded symbols) and controls (open symbols) and then compared prevalence between cases who had consumed milk and cases who had consumed low‐galactose formula in the 1st year of life

Our first analysis addressed the prevalence of acute complications in early infancy between cases and controls (Figure 1). This was an important point to test, given the prevalence of acute health complications experienced by infants with classic galactosemia following exposure to either breast milk or dairy milk–based formula.^2^ Of children in our cohort for whom the relevant data were available, 52 of 204 cases (26.2%) and 31 of 143 controls (22.2%) reported experiencing some acute complications. This difference was not significant (*p* = 0.587), and of potential covariates tested (age, sex, race, relative family income, and parent education level), none were significant.

Next, we asked whether children with DG who had consumed milk as infants experienced a higher prevalence of acute complications than their counterparts who had consumed low‐galactose formula. We found that 21 of 83 (25.3%) cases who drank milk and 31 of 121 (25.6%) cases who drank low‐galactose formula reported acute complications. As above, these differences were not significant (*p* = 0.986) and again, none of the potential covariates tested were significant. Of note, the rates and types of neonatal complication reported in our cohort were at or below the levels reported previously for a general population.^7^

### 3.3 Receipt of early intervention services when <3 years old

We found that 12 of 206 cases (5.8%), and 5 of 144 controls (3.5%), had received early intervention services when <3 years old and the remainder had not. As above, these differences were not significant (*p* = 0.179), and none of the covariates tested were significant. With regard to infant diet, 3 of 83 (3.6%) cases who had consumed milk and 9 of 123 (7.32%) cases who had consumed low‐galactose formula had received early intervention services. Again, this difference was not statistically significant (*p* = 0.275). In testing possible covariates, only race was significant (*p* = 0.0425), with children identified by their parents as “White, non‐Hispanic” less likely to have received early intervention services. However, because the D2 *GALT* allele that underlies DG is found almost exclusively in populations of European ancestry,^8^ the percentage of children identified as “White, non‐Hispanic” in this study was overwhelming (>93%), raising concern that any difference associated with race might be an artifact of small numbers in one comparison group.

### 3.4 Receipt of special educational services when 3–5 years old

We found that 9 of 206 (4.4%) cases and 6 of 144 (4.2%) controls had received special educational services when 3–5 years old. As above, this difference was not significant (*p* = 0.941). For receipt of services when 3–5 years old, sex was a significant covariate (*p* = 0.021), with boys more likely than girls to have received special services. Since boys and girls were both well represented in our study cohort, this is likely to be a meaningful difference and indeed mirrors what is seen in the general population.^9^ With regard to infant diet, 2 of 83 (2.4%) cases who had consumed milk, and 7 of 116 (5.7%) cases who had consumed low‐galactose formula, had received special services. While notable (see Section 4), this difference was not significant (*p* = 0.203) and no covariates were significant in this analysis.

## 4 DISCUSSION

Previously, we reported the results of direct developmental assessments of a cohort of 206 children with DG and 144 unaffected sibling controls, all 6–12 years old at the time of testing. In that study, we found no significant differences between cases and controls in the prevalence of detectable developmental complications and also no significant association among cases between developmental outcomes in midchildhood and dietary exposure to galactose in the 1st year of life.^4^

Here, we extend from those results, addressing infant health and receipt of early intervention and/or special educational services up to Age 5 for the same cohort of children. Specifically, we found no significant differences between cases and controls in parent/guardian reports of the prevalence of (1) acute complications in infancy, (2) having received early intervention services when <3 years old, or (3) having received special educational services when 3–5 years old. We also found no significant differences among cases for any of these three outcome measures as a function of dietary exposure to galactose in the 1st year of life.

For both receipt of early intervention when <3, and receipt of special educational services when 3–5 years old, we actually found a greater difference between galactose‐restricted cases and untreated cases than between cases and controls. While none of these differences were statistically significant, they were nonetheless striking because for both variables, cases who had received dietary intervention as infants were more likely, not less likely, to have also received early intervention and/or special educational services. To be clear, this is opposite to the pattern we would have expected were milk exposure in infancy a risk factor predisposing children with DG to developmental complications later in childhood. Combined, these results support the conclusion that DG is a benign condition for infants and children and that dietary restriction of galactose does not benefit the detectable outcomes of children with DG in infancy, early childhood, or as previously demonstrated,^4^ in midchildhood. Indeed, that treated cases reported an approximately 2‐fold higher prevalence of receiving both early intervention and special educational services compared with their untreated DG counterparts (7.32% vs. 3.6% and 5.7% vs. 2.4%, respectively) is a trend worth noting. It may suggest that the process of requiring parents to restrict their baby's dietary galactose exposure in the 1st year of life has lasting effects on parental concern about their child's later developmental outcomes; for example, motivating them to seek special educational services.

### 4.1 Strengths and limitations

The study reported here had four main strengths: a large cohort size, the outcome‐neutral manner in which families were recruited,^4^ a broad, albeit US‐only geographic range, and the inclusion of a well‐matched internal control group. This study also had limitations, perhaps the most notable being that our neonatal and early childhood outcome data, as well as infant diet data, were gathered by retrospective parent/guardian report rather than direct assessment. That said, from the entirety of the survey responses we received, it was clear that the parent/guardians in our study had very detailed recollections of their child's infancy and early years.

A further limitation was the indirect nature of the data we collected with regard to child developmental outcomes. Specifically, receipt of special services, especially for very young children, may be either preventative or responsive and is therefore an imperfect proxy for actual child outcome. As mentioned above, that the cases in our study who had received dietary intervention as infants were close to twice as likely as their untreated DG counterparts to have received early intervention and/or special educational services may speak to this point.

### 4.2 Comparison with prior studies

Two prior studies also addressed developmental outcomes in very young children with DG. The first, published in 2008 by Ficicioglu and colleagues,^10^ reported the results of direct assessments of 28 children with DG, most 1–3 years old at the time of testing; some had consumed milk and others low‐galactose formula. All scored within the normal range for general cognitive ability, language development, and adaptive functioning.^10^ A second report, published in 2021 by Waisbren and colleagues,^11^ presented the results of a hospital medical records review that revealed 21 children with DG, average age 3 years; again, some had consumed milk and others had consumed low‐galactose formula. As in the earlier study, all of these children had developmental test scores within the normal range, but eight (42%) had participated in early intervention and/or special education and six (32%) had received speech therapy. Whether these interventions were considered preventative, or responsive, was not reported, but the authors did note that those tested at age < 1 year old, but not >1 year old, showed speech/language skills within normal limits but a relative weakness in motor development compared with cognitive development. The significance and implications of that observation remain unclear.

## 5 CONCLUSION

The results reported here address two of the previously unresolved concerns about outcomes of young children with DG, namely risk of acute health complications in infancy following milk exposure and risk of early and possibly transient developmental delays in the early years.^11^ To the limits of our study, we counter both of these concerns.

## CONFLICT OF INTEREST

The authors declare that they have no competing interests.

## AUTHOR CONTRIBUTIONS

Judith Fridovich‐Keil initiated the project, coordinated the efforts of all coauthors, created the figure, and wrote most of the manuscript. Grace Carlock was instrumental in collecting and organizing the data, performed early data analyses, and assisted with editing the manuscript. Sneh Patel performed all statistical analyses presented and contributed to writing and editing of the manuscript. Nancy Potter was instrumental in collecting the data and assisted with editing the manuscript. Claire Coles contributed to gathering and interpreting the data and editing the manuscript.

## ANIMAL RIGHTS

This study did not involve laboratory animal subjects.

## ETHICS STATEMENT

All human subjects work reported here was approved by the Emory University Institutional Review Board as part of protocol 00081271 (PI: JL Fridovich‐Keil).

## PATIENT CONSENT STATEMENT

All volunteers participated following appropriate informed consent (and assent, for pediatric volunteers) in Emory IRB protocol 00081271.

## Data Availability

The manuscript has no associated data.

## References

[1] Fridovich‐Keil JL , Gambello MJ , Singh RH , Sharer JD . Duarte variant galactosemia. In: Adam MP , Ardinger HH , Pagon RA , et al., eds. GeneReviews((R)). University of Washington, Seattle; 1993.

[2] Berry GT . Classic galactosemia and clinical variant galactosemia. In: Pagon RA , Adam MP , Ardinger HH , et al., eds. GeneReviews(R). University of Washington, Seattle; 1993.

[3] Pyhtila BM , Shaw KA , Neumann SE , Fridovich‐Keil JL . Newborn screening for galactosemia in the United States: looking back, looking around, and looking ahead. JIMD Rep. 2015;15:79‐93. 24718839 10.1007/8904_2014_302 PMC4413015

[4] Carlock G , Fischer ST , Lynch ME , et al. Developmental outcomes in Duarte Galactosemia. Pediatrics. 2019;143:e20182516. 30593450 10.1542/peds.2018-2516

[5] Harris PA , Taylor R , Minor BL , et al. The REDCap consortium: building an international community of software platform partners. J Biomed Inform. 2019;95:103208. 31078660 10.1016/j.jbi.2019.103208 PMC7254481

[6] Harris PA , Taylor R , Thielke R , Payne J , Gonzalez N , Conde JG . Research electronic data capture (REDCap)—a metadata‐driven methodology and workflow process for providing translational research informatics support. J Biomed Inform. 2009;42:377‐381. 18929686 10.1016/j.jbi.2008.08.010 PMC2700030

[7] Woodgate P , Jardine LA . Neonatal jaundice. BMJ Clin Evid. 2011;2011:0319. PMC3217664 21920055

[8] Carney AE , Sanders RD , Garza KR , et al. Origins, distribution and expression of the Duarte‐2 (D2) allele of galactose‐1‐phosphate uridylyltransferase. Hum Mol Genet. 2009;18:1624‐1632. 19224951 10.1093/hmg/ddp080 PMC2667289

[9] Oswald DP , Best AM , Coutinho MJ , Nagle HAL . Trends in the special education identification rates of boys and girls: a call for research and change. Exceptionality. 2003;11:223‐237.

[10] Ficicioglu C , Thomas N , Yager C , et al. Duarte (DG) galactosemia: a pilot study of biochemical and neurodevelopmental assessment in children detected by newborn screening. Mol Genet Metab. 2008;95:206‐212. 18976948 10.1016/j.ymgme.2008.09.005

[11] Waisbren SE , Tran C , Demirbas D , et al. Transient developmental delays in infants with Duarte‐2 variant galactosemia. Mol Genet Metab. 2021;134:132‐138. 34391645 10.1016/j.ymgme.2021.07.009

